# Persistent Mosaicism for 12p Duplication/Triplication Chromosome Structural Abnormality in Peripheral Blood

**DOI:** 10.1155/2013/857926

**Published:** 2013-09-15

**Authors:** Amy L. Shackelford, Laura K. Conlin, Marybeth Hummel, Nancy B. Spinner, Sharon L. Wenger

**Affiliations:** ^1^Department of Pathology, West Virginia University, Morgantown, WV 26506-9203, USA; ^2^Department of Human Genetics, University of Pennsylvania, Philadelphia, PA 19104, USA; ^3^Department of Pediatrics, West Virginia University, Morgantown, WV 26506-9214, USA

## Abstract

We present a rare case of mosaicism for a structural abnormality of chromosome 12 in a patient with phenotypic features of Pallister-Killian syndrome. A six-month-old child with dysmorphic features, exotropia, hypotonia, and developmental delay was mosaic for both a normal karyotype and a cell line with 12p duplication/triplication in 25 percent of metaphase cells. Utilization of fluorescence in situ hybridization (FISH) identified three copies of probes from the end of the short arm of chromosome 12 (TEL(12p13) locus and the subtelomere (12p terminal)) on the structurally abnormal chromosome 12. Genome-wide SNP array analysis revealed that the regions of duplication and triplication were of maternal origin. The abnormal cell line in our patient was present at 25 percent at six months and 19 months of age in both metaphase and interphase cells from peripheral blood, where typically the isochromosome 12p is absent in the newborn. This may suggest that the gene(s) resulting in a growth disadvantage of abnormal cells in peripheral blood of patients with tetrasomy 12p may not have the same influence when present in only three copies.

## 1. Introduction 

Patients with trisomy 12p typically present with severe mental retardation, seizures, low-set ears, and characteristic facial dysmorphology including flatness of the face, small nose with broad bridge, anteverted nares, inner epicanthal folds, long philtrum, everted lower lip, and high forehead. The extra copy is due to an intrachromosomal duplication or an extra copy on a derivative chromosome. Patients with tetrasomy 12p, or Pallister-Killian syndrome (PKS), additionally present with sparse temporal hair, eyebrows, and eyelashes, prominent forehead, a cupid-bow shaped mouth, and large ears. A hallmark of PKS is tissue-limited mosaicism, with few, if any, abnormal cells found in peripheral blood lymphocyte metaphases in the newborn. Another characteristic of tetrasomy 12p is loss of the abnormal cell line in peripheral blood and skin fibroblasts as the patient ages or over time in serial-passaged cultured fibroblasts [1, 2].

We report on a patient with mosaicism for duplication and triplication of 12p. Only about 24 cases with mosaicism for a structural abnormality of an autosome have been reported in the literature [3]. Approximately 26 individuals with nonmosaic structural 12p duplications have been reported in the literature with a minimum critical region of 12p13.31 [4]. We report on a patient with mosaicism in peripheral blood for a derivative chromosome involving duplication and triplication of 12p.

## 2. Clinical Report

Our patient was delivered at 38 weeks gestation to a 19-year-old mother. The pregnancy was complicated by gestational diabetes and polyhydramnios. At birth, the infant presented with an anal fistula, hypertension, mild hypotonia, ventricular septal defect (VSD), and intraventricular hemorrhage and was hospitalized for 3 and a half weeks due to breathing difficulties. At 5 months of age, the patient was able to roll over and sit with support and had exotropia. She was seen by genetics at 6 months of age and was found to be dysmorphic and hypotonic with significant developmental delays. At ten months, she was unable to crawl or sit without support. Her height and weight were in the 50th percentile, with head circumference at the 90th percentile. At 19 months, the patient had a broad, high forehead, bitemporal balding, small posteriorly rotated ears, global developmental delays, and mild hypotonia. She could sit but not pull to a stand. She was asymptomatic for VSD, had eye surgery to remove chalazia, and was receiving physical, speech, and vision therapies as well as seeing a developmental specialist.

## 3. Materials and Methods

Peripheral blood, obtained from our patient and her parents, was processed using routine cytogenetic procedures to obtain a karyotype and a DNA extraction for microarray analysis. FISH was performed on the patient's peripheral blood metaphases using the centromere 12, TEL (12p13), and subtelomere 12p probes from Vysis (Abbott Molecular, Inc., Downers Grove, IL). All probes were diluted (2 *μ*L : 50 *μ*L) in *c*DenHyb (InSitus Biotechnologies, Albuquerque, NM) and hybridized to the target locations on chromosome 12 following the manufacturer's protocol. One hundred cells were scored on each probe to determine the percentages of normal and abnormal cells.

Genome-wide SNP array analysis using Illumina Quad 610 array was performed in the Cytogenomics Laboratory at the Children's Hospital of Philadelphia on the extracted DNA. The array contains 28,528 SNP probes on chromosome 12. Log *R* ratios were used to determine the dosage of patient DNA by intensity of signal, and parental DNA was analyzed to determine the origin of the duplicated chromosomal segment in the child. B allele frequency was calculated using genotype clusters per SNP as determined from HapMap sample analysis. Methods for SNP array analysis were as previously described, and mosaicism of 20% was computed based on the B allele frequencies [5, 6].

## 4. Results

**Figure 1:**
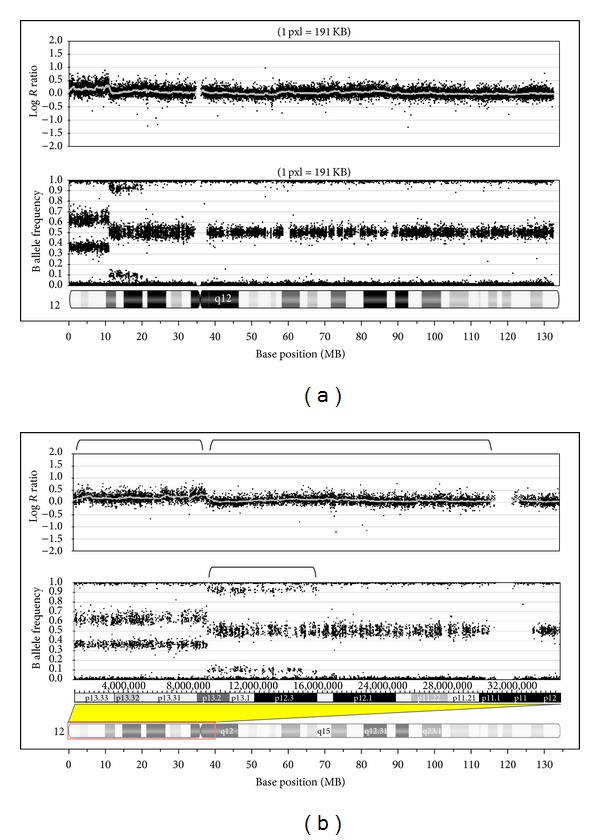
(a) SNP array results for chromosome 12 showing Log *R* ratios in the top panel and B allele frequency in the bottom panel. The long arm of chromosome 12 shows no copy number of genotyping abnormalities. The short arm shows two regions of copy number change, with more copies of the terminal region of 12p and the proximal 12p region. (b) SNP array results for 12p only with the Log *R* ratio in the upper panel and the B allele frequency in the bottom panel. Regions of mosaicism for four copies (terminal) and three copies (proximal) are indicated by brackets. The additional genotypes in the region of mosaicism for three copies are shown by the bracket in the lower panel. This genotyping pattern indicates that the extra copy of 12p in this region contains an additional maternal haplotype. The presence of three haplotypes suggests an origin of the abnormal 12p in meiosis.

**Figure 2:**
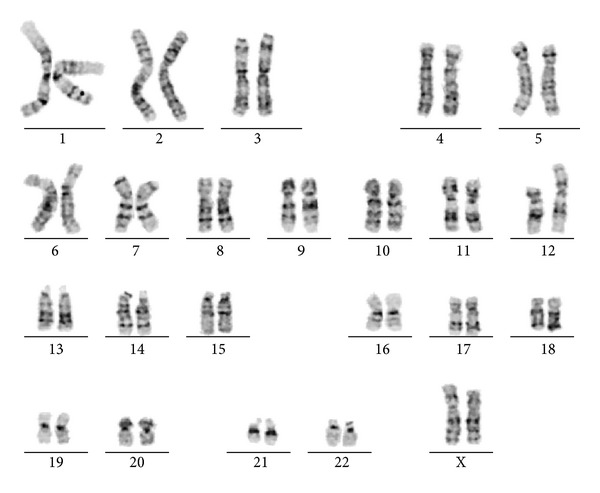
Karyotype of 46,XX,dup(12)(p11.2p13.2),trp(12)(p13.2 pter) seen in 25% of peripheral blood metaphase cells.

FISH studies confirmed four copies of the TEL (12p13) and subtelomere (12p terminal) probes. SNP array analysis indicated that there were 3 copies of 12p11.21 to 12p13.2 with three haplotypes for 12p11.2 to 12p13.2. The additional material was identified as maternal in origin through the use of informative SNPs and comparison of parent and child genotypes. At least four copies of 12p13.2 to 12pter were identified with two haplotypes (Figure 1). Our patient's karyotype was interpreted as 46,XX,dup(12)(p11.2p13.2),trp(12)(p13.2pter)[5]/46,XX[15]; twenty-five percent of her cells had three to four copies of 12p (Figure 2); the other seventy-five percent were normal.

## 5. Discussion

 Approximately 26 individuals with varying 12p structural duplications or triplications have appeared in the literature [4], most shared phenotypic features found in PKS. These similarities in the clinical presentation of our patient to PKS suggested the likelihood of the abnormal cell line completely disappearing from peripheral blood as our patient ages, as tissue-limited mosaicism is a hallmark of PKS presentation. Previously reported cases with 12p duplications (three copies) [4] were present in all peripheral blood cells. Two cases with triplications for all of 12p showed tissue limited mosaicism, with the abnormal cell line being present in only skin fibroblasts [7, 8]. However, two cases with triplication of 12p regions that did not include 12p13.31 were present in all tissues, including peripheral blood [9, 10]. Our patient has four copies of the region proposed to be responsible for the PKS phenotype, 12p13.31, which contains three genes, ING4, CHD4, and MAGP2, responsible for negative growth regulation [4]. Overexpression of ING4 has been shown to result in cell cycle arrest [11].

Genome-wide SNP array analysis identified three copies of 12p13.2 to 12p11.21 and confirmed the presence of four copies of 12pter to 12p13.2. The additional material was found to be maternal in origin through the use of informative SNPs in the parents (AA v BB). The presence of both a normal cell line and an abnormal cell line with a structural abnormality suggests a mitotic error. However, the SNP results indicated maternal meiotic crossing over, consistent with nondisjunction in meiosis after the crossing over occurred. There are new genotype patterns in the patient from 12p12.2 to 12p13.2, and the remainder of the abnormal 12p arm had triplication of the maternal chromosomal material. This may have occurred during meiosis II, due to the lack of extra genotypes near the centromere. The report of two patients with mosaicism for de novo duplications identified a meiotic error and proposed two trisomy rescue events during mitotic divisions early during embryogenesis [12].

Izumi and colleagues [4] reported that the critical region for PKS is 12p13.31 based upon a case with an interstitial duplication of 12p and a review of the literature. Our patient had four copies of this region and a PKS phenotype, as expected. However, our patient only has three copies of the 12p11.2 to 12p13.2 region, as opposed to the tetrasomy seen with the isochromosome 12p. While the isochromosome 12p marker is rarely seen in peripheral blood, the abnormal cell line in our patient is present in twenty-five percent of her peripheral blood cells at six and 19 months of age. The isochromosome 12p marker has been reported to be at a higher percentage in interphase than in metaphase cells from patients with PKS [13]. Our patient, however, has the same percentage of the abnormal cell line in both metaphase and interphase cells. The lack of change in mosaicism in our patient may suggest that the gene(s) responsible for growth disadvantage in peripheral blood may be located outside the region present in four copies in our patient. Since our patient has at least four copies of the 12p13.31 critical region, this might suggest that the genes that affect cell survival in peripheral blood may be proximal to the 12p13.31 region, which would explain the stability of the abnormal cell line in our patient. This will need to be confirmed by the identification of other PKS patients with mosaicism in peripheral blood.
